# Facial Burn Healing With a Polylactic Acid Dermal Matrix: A Case Report on Wound Modulation and Graft-Free Epithelialization

**DOI:** 10.1093/jbcr/iraf127

**Published:** 2025-07-08

**Authors:** Mario Aurelio Martínez-Jiménez, Ana Lorena Novoa-Moreno, Rodolfo Ariel Miranda-Altamirano, Olga Johnson-Ponce, Eleazar Samuel Kolosovas-Manchuca, Victor Manuel Loza-González

**Affiliations:** Burn Unit, Hospital Regional de Alta Especialidad “Ignacio Morones Prieto”, San Luis Potosí, San Luis Potosí 78290, México; Burn Unit, Hospital Regional de Alta Especialidad “Ignacio Morones Prieto”, San Luis Potosí, San Luis Potosí 78290, México; Unidad de Atención Integral a Niños, Niñas y Adolescentes con Quemaduras, Antiguo Hospital Civil de Guadalajara “Fray Antonio Alcalde”, Guadalajara, Jalisco 45425, México; Department of Pathology, Hospital Regional de Alta Especialidad “Ignacio Morones Prieto”, San Luis Potosí, San Luis Potosí 78290, México; Coordinación para la Innovación y Aplicación de la Ciencia y la Tecnología, Universidad Autónoma de San Luis Potosí, San Luis Potosí, San Luis Potosí 78210, México; Doctorado Institucional en Ingeniería y Ciencias de Materiales, Universidad Autónoma de San Luis Potosí, San Luis Potosí, San Luis Potosí 78210, México

**Keywords:** facial chemical burns, polylactic acid, burn wound management, case report

## Abstract

Due to their functional and aesthetic implications, facial burns pose significant clinical challenges. Alkali burns can further complicate these injuries by causing deep tissue necrosis, which complicates healing and increases the risk of scarring. Traditional management involves early excision and autografting, but challenges such as donor site morbidity and poor aesthetic integration remain. SUPRA SDRM, a fully synthetic, polylactic acid (PLA)-based resorbable dermal matrix, has demonstrated efficacy in different wound types but has not been previously reported in deep burn management. Here, we present the case of a 53-year-old male with deep alkali facial burns managed with SUPRA SDRM. Initially applied as a bridge to grafting, rapid pain relief, and early vascularization led to an alternative treatment course. The matrix formed an adherent synthetic wound barrier, modulating the healing environment and allowing full epithelialization within 5 weeks without the need for grafting. Histology confirmed enhanced angiogenesis and dermal remodeling, while infrared thermography imaging demonstrated changes suggestive of perfusion improvements, which supports the bio-inductive effects of the matrix. This case suggests that PLA-based dermal matrices may serve as a viable alternative to autografting in select deep burns, warranting further investigation into their role in wound modulation, functional outcomes, and resource-limited burn care.

## INTRODUCTION

Facial burns present a unique clinical challenge due to their functional, aesthetic, and psychological consequences. These injuries can cause contractures and disfigurement that impair essential functions such as vision, speech, mastication, facial expression, and communication; factors that significantly impact quality of life and are major sources of distress among survivors.^1^ Although the rich vascularity of facial tissue supports rapid healing, it also predisposes patients to excessive inflammation, hypertrophic scarring, and pigmentation abnormalities. Thus, minimizing disfigurement and preserving function are key goals in facial burn management.^2^

Injuries caused by alkalis are particularly severe, as they induce liquefaction necrosis with deep tissue penetration and ongoing cellular damage, often resulting in more extensive injury than other burn types. Alarmingly, alkalis have recently emerged as agents of assault, increasing the prevalence of these burns in facial trauma.^3^ Standard treatment for deep facial burns includes early excision and autografting to reduce infection risk, scarring, and delayed healing. However, it poses challenges such as donor site morbidity, color mismatch, and visible textural differences.^4^ Skin substitutes have shown promise in enhancing wound healing, scar quality, and patient comfort, although they may slightly delay early re-epithelialization.^5^

Here, we present the case of a patient with a full-thickness alkali facial burn treated with SUPRA SDRM, a synthetic, biodegradable polylactic acid (PLA) matrix (Polymedics Innovations, Kirchheim unter Teck, Germany),^6^ to optimize wound bed preparation. Following the patient’s refusal of autografting, reapplication of the PLA matrix resulted in complete wound closure without the need for grafting.

## CASE PRESENTATION AND RESULTS

**Figure 1:**
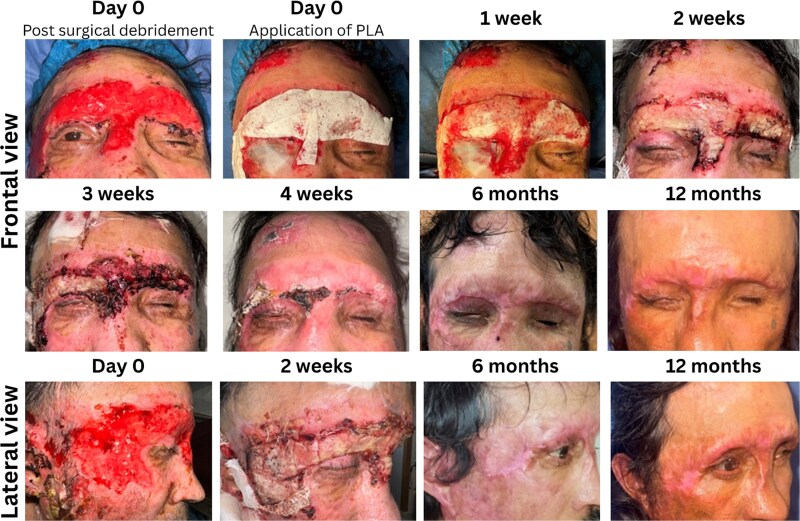
Clinical Progression of a Facial Chemical Burn Following Treatment • Top Row (Left to Right): Initial Appearance Immediately After Surgical Debridement, Followed by Application of a Polylactic Acid Matrix on the Same Day. Then, Wound Progression at 1 and 2 Weeks Postapplication • Middle Row (Left to Right): Continued Healing at 3 Weeks and 4 Weeks, With the Latter Demonstrating Full Wound Closure. Next, Follow-Up Images at 6 and 12 Months, Highlighting Long-Term Outcomes • Bottom Row (Left to Right): Lateral View of the Wound, Beginning With the Initial Presentation, Progression at 4 Weeks, and Concluding With 6- and 12-Month Follow-Up Images

A 53-year-old man experiencing homelessness presented to our burn unit 48 h after an alkali-based chemical assault to the face. He received initial decontamination at a community hospital before transfer for specialized care. On admission, he was hemodynamically stable but reported severe pain (9/10). Examination revealed complete epidermal and dermal loss, sparing the eyes but involving the right upper eyelid; the left eye had a prior enucleation (Figure 1).

**Figure 2:**
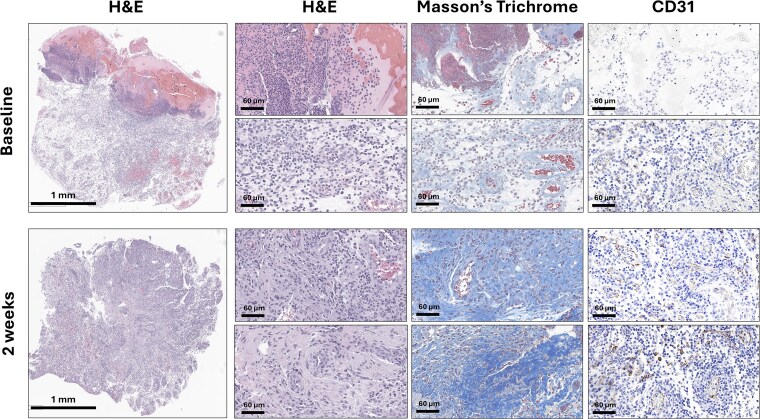
Left Eyebrow Biopsies Taken at Baseline (Pretreatment) and 2 Weeks Posttreatment Low-Magnification Views of 3 mm Punch Biopsies Stained With Hematoxylin and Eosin (H&E), Massons’s Trichrome, and CD31 for Endothelium Confirm a Full-Thickness Alkali Burn Characterized by Extensive Liquefaction Necrosis, Dense Inflammatory Infiltrates, Disrupted Collagen Architecture, and Capillary Microthrombosis With Endothelial Wall Destruction Two Weeks Posttreatment; H&E Staining Reveals Reduced Inflammatory Infiltrates, on Masson’s Trichrome Staining Abundant Extracellular Matrix Deposition, With Randomly Oriented Collagen Fibrils, Indicative of Early Neo-dermis Formation. CD31 Staining Highlights a Robust Neo-angiogenic Response.

**Figure 3:**
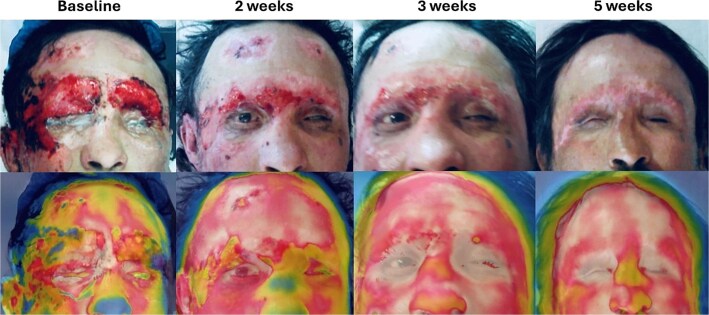
Infrared Thermographic Assessment At Baseline, Thermography Shows Hypothermic Regions (Δ*T* < −3.5°C) in Burned Areas. By 2 Weeks, Δ*T* Narrows to ≈ −2°C With Emerging Hotspots at Wound Margins, Suggesting Improved Vascularization and Active Epithelialization. At 3 Weeks, Thermal Asymmetry Continues to Decrease. By Week 5, Thermograms Resemble Uninjured Skin, No Hotspots or Coldspots, Indicating Resolved Inflammation and Restored Microvascular Function.

Tangential excision using the Versajet hydrosurgery system was performed for wound bed optimization. Histology confirmed mixed partial and full-thickness burns with necrosis, edema, and vascular injury (Figure 2). Digital planimetry (Swift Medical, Toronto, Canada) measured the burnt area at 38.5 cm^2^, affecting functionally and cosmetically critical facial regions. Infrared thermography (IRT) scan using a FLIR T600 camera (Teledyne FLIR, Wilsonville, OR) showed Δ*T* > −3.5°C compared to adjacent skin, indicating poor healing potential and supporting histopathologic findings (Figure 3).^7–9^

SUPRA SDRM was applied intraoperatively to optimize the wound bed for autologous split-thickness skin grafting (STSG) at day 14 postinjury. Due to the strong integration of SDRM into the wound bed and the development of a dry, adherent protective layer, the matrix was left uncovered. Pain reduced rapidly to 2/10 within 24 h, allowing discontinuation of opioids.

At 2 weeks, advancing epithelial margins and complete epithelialization of the right upper eyelid without contracture were observed. Infrared thermography imaging showed reduced Δ*T* values, and histopathological analysis revealed new collagen deposition, reduced inflammatory infiltrate with macrophage predominance, and increased angiogenesis evidenced by CD31-positive endothelial cells forming capillary structures (Figure 2). Despite a scheduled STSG, the patient declined further surgery due to minimal symptoms. Instead, a second SDRM application was performed at bedside.

By week 3, 50% of the wound was epithelialized, with the PLA matrix detaching from the fully healed areas and a third SDRM application was performed. By week 4, 80% of the burn was re-epithelialized, prompting discharge into the general ward. On the fifth and final week, a complete epithelialization and temperature symmetry with the IRT, showed a complete healing^10^ (Figure 2). Due to these findings the patient was discharged from the hospital with instructions for outpatient follow-up.

At a 3-, 6-, and 12-month follow-up posttreatment, the patient showed stable healing with mild hypopigmentation, preserved facial mobility, no inflammation, and no signs of contracture or hypertrophic scarring (Figure 1).

## DISCUSSION

This case highlights the successful use of a PLA-based dermal matrix in managing a full-thickness chemical facial burn, achieving full healing without autografting, while also providing effective pain relief and reducing inflammation. The decision to use SUPRA SDRM was based on prior studies demonstrating favorable outcomes with PLA-based membranes.^6^^,^^11^^,^^12^

The clinical efficacy observed can be attributed to the matrix’s dual role as a physical scaffold and a bioactive modulator. It integrated into the wound bed, forming a synthetic barrier that retained moisture, reduced contraction, and protected exposed nerve endings. Concurrently, lactate released from the matrix lowered local pH, providing antimicrobial effects,^13^^,^^14^ and promoted wound healing through pro-angiogenic, anti-inflammatory, and ECM-stimulating pathways.^15–17^ Histopathologic findings at 2 weeks supported this hypothesis, showing neo-dermis–like structures, collagen deposition, reduced inflammation, and robust capillary formation, suggesting active participation of the matrix in tissue regeneration.

Another novel aspect of this case was the use of IRT as an objective assessment tool, enabling real-time evaluation of tissue perfusion and healing progression.^7^^,^^9^^,^^10^

While the outcomes are promising, this report is inherently limited by its single-patient design. Further studies are needed to evaluate reproducibility, compare efficacy with conventional treatments and assess long-term durability of the regenerated tissue. Outcomes such as scar maturation and resilience to re-injury over time remain to be fully understood. Nonetheless, based on serial follow-up, the regenerative effects appear sustained.

## CONCLUSION

This case highlights the potential of alloplastic skin substitutes to shift the paradigm in burn care by offering a non-invasive alternative to traditional multi-stage reconstruction. The successful use of a fully synthetic dermal matrix to achieve deep burn healing without grafting opens new possibilities for resource-limited settings, mass casualty incidents, or when surgical options are declined, balancing effective healing with minimal intervention. Looking ahead, integrating bio-inductive materials, real-time perfusion tools, and patient-centered models may help redefine standards in burn and complex wound care.

Patient gave written consent of using his clinical case to be shown in this study.

## Notes

Author contributions: Mario Aurelio Martinez-Jimenez (Conceptualization [lead], Data curation [equal], Supervision, Writing—review & editing [lead]), Ana Lorena Novoa-Moreno (Data curation [supporting], Writing—original draft [lead], Writing—review & editing [equal]), Rodolfo Ariel Miranda-Altamirano (Visualization, Writing—review & editing [supporting]), Olga Johnson-Ponce (Formal analysis, Validation [equal]), Eleazar Samuel Kolosovas-Manchuca (Formal analysis, Software [equal]), and Victor Manuel Loza-González (Formal analysis, Methodology [equal]).

Funding: We have not received any funding, fees, or benefits from any commercial source or other entity that could influence the results and conclusions presented in this work.
